# Modulation of Distinct Asthmatic Phenotypes in Mice by Dose-Dependent Inhalation of Microbial Products

**DOI:** 10.1289/ehp.1307280

**Published:** 2013-10-29

**Authors:** Gregory S. Whitehead, Seddon Y. Thomas, Donald N. Cook

**Affiliations:** Laboratory of Respiratory Biology, National Institute of Environmental Health Sciences, National Institutes of Health, Department of Health and Human Services, Research Triangle Park, North Carolina, USA

## Abstract

Background: Humans with asthma display considerable heterogeneity with regard to T helper (Th) 2–associated eosinophilic and Th17-associated neutrophilic inflammation, but the impact of the environment on these different forms of asthma is poorly understood.

Objective: We studied the nature and longevity of asthma-like responses triggered by inhalation of allergen together with environmentally relevant doses of inhaled lipopolysaccharide (LPS).

Methods: Ovalbumin (OVA) was instilled into the airways of mice together with a wide range of LPS doses. Following a single OVA challenge, or multiple challenges, animals were assessed for pulmonary cytokine production, airway inflammation, and airway hyperresponsiveness (AHR).

Results: Mice instilled with OVA together with very low doses (≤ 10^–3^ μg) of LPS displayed modest amounts of Th2 cytokines, with associated airway eosinophilia and AHR after a single challenge, and these responses were sustained after multiple OVA challenges. When the higher but still environmentally relevant dose of 10^–1^ μg LPS was used, mice initially displayed similar Th2 responses, as well as Th17-associated neutrophilia. After multiple OVA challenges, however, the 10^–1^ μg LPS animals also accumulated large numbers of allergen-specific T regulatory (Treg) cells with high levels of inducible co-stimulatory molecule (ICOS). As a result, asthma-like features in these mice were shorter-lived than in mice sensitized using lower doses of LPS.

Conclusions: The nature and longevity of Th2, Th17, and Treg immune responses to inhaled allergen are dependent on the quantity of LPS inhaled at the time of allergic sensitization. These findings might account in part for the heterogeneity of inflammatory infiltrates seen in lungs of asthmatics.

Citation: Whitehead GS, Thomas SY, Cook DN. 2014. Modulation of distinct asthmatic phenotypes in mice by dose-dependent inhalation of microbial products. Environ Health Perspect 122:34–42; http://dx.doi.org/10.1289/ehp.1307280

## Introduction

Allergic asthma is a chronic disease of the airways characterized by airway inflammation, mucus production, and reversible airway obstruction (Busse and Lemanske 2001). These features stem largely from the actions of T helper (Th) 2 cells, which produce the cytokines interleukin (IL)-4, IL-5, and IL-13 (Herrick and Bottomly 2003; Larche et al. 2003) and drive eosinophilic inflammation and mucus production. However, individuals with asthma display considerable heterogeneity with regard to inflammatory cells in the airway, and many patients have neutrophilic inflammation with little evidence of eosinophilia (McGrath et al. 2012). Unfortunately, these patients respond poorly to inhaled corticosteroids, the standard asthma therapy, and emerging data suggest that neutrophilic asthma might result from IL-17A (IL-17) production by steroid-resistant Th17 cells (Barczyk et al. 2003; Bullens et al. 2006; McKinley et al. 2008). Although IL-17 does not directly recruit neutrophils, it triggers airway epithelial cells to secrete neutrophil-attracting chemokines, such as IL-8 (Laan et al. 1999). In addition to having allergen-specific Th2 and Th17 effector responses, asthmatics also appear to lack sufficiently strong regulatory mechanisms to keep effector responses in check (Akdis et al. 2004; Lloyd and Hawrylowicz 2009).

The increased prevalence of allergic asthma over the last several decades has been linked to exposure to several environmental factors, including bacteria and their products. Accordingly, the “hygiene hypothesis” postulates that increased hygiene, smaller families, and consequent decreased exposure to pathogens is at least partially responsible for the increase in allergic diseases (Strachan 1989). It was initially proposed that infections protect against Th2-related diseases by skewing immunity toward Th1 responses, but more recent evidence suggests that increased regulatory responses are responsible for this protective effect (Yazdanbakhsh et al. 2002). It is currently unclear whether the quantities of bacterial products in natural environments affect the balance of effector and regulatory responses to inhaled allergens.

Lipopolysaccharide (LPS), a major component of the outer cell wall of gram-negative bacteria, is ubiquitous in the environment, and some studies have indicated that exposure to relatively high levels of LPS during childhood protect against developing asthma later in life (Braun-Fahrländer et al. 2002; El-Sharif et al. 2006; Gehring et al. 2004). Conversely, other studies have demonstrated a positive association between asthma and household levels of bacteria and LPS (Ross et al. 2000; Thorne et al. 2005). Eisenbarth et al. (2002) previously reported that in mice, a large amount of inhaled LPS (10 μg) promotes Th1 responses, whereas a smaller amount (100 ng) promotes Th2 responses. Subsequent studies showed that LPS can also promote Th17 responses in the lung (Wilson et al. 2009) and intestine (McAleer et al. 2010). Paradoxically, LPS can also induce T-regulatory (Treg) cell expansion directly (Caramalho et al. 2003) or indirectly by activating IL-10–producing dendritic cells (Lau et al. 2008). In most of these experiments, relatively large amounts of LPS were used, and the impact of more environmentally relevant amounts of LPS on immune responses is unclear. Finally, it is not known whether inhaled LPS affects immune responses to intermittent allergen exposures or responses to chronic exposures such as those occurring in the home or work environments. Accordingly, the present study was initiated to test the hypothesis that different amounts of inhaled LPS can not only trigger different forms of asthma but also modulate regulatory responses that control the actions of allergen-specific effector T cells. We used established mouse models of asthma based on a highly purified form of ovalbumin (OVA), which lacks detectable LPS and is nonallergenic in the absence of exogenously added adjuvants (Eisenbarth et al. 2002; Wilson et al. 2009). One model mimicked intermittent exposures and the other mimicked more chronic exposures. We observed that extremely low doses of inhaled LPS are sufficient to prime immune responses to OVA, and that these responses lead to sustained airway eosinophilia and airway hyperresponsiveness (AHR). Higher—but still environmentally relevant—doses of LPS induced both Th2 and Th17 responses in regional lymph nodes (LNs). In addition, regulatory responses were induced, and as a result, asthma-like responses in these animals were consequently short-lived. Thus, we found that levels of microbial products determined the balance of Th2, Th17, and regulatory responses to either promote or suppress the asthmatic phenotype. We also observed that the amounts of LPS found naturally in house dust are sufficient to confer both Th17 and Treg responses in a dose-dependent manner. These findings suggest that differing levels of environmental LPS in previous studies might explain discordant conclusions regarding the effect of environmental endotoxin on allergic responses.

## Materials and Methods

*Mice.* C57BL/6J (carries CD45.2), BALB/​cJ, B6.Cg-*Foxp3^tm2(EGFP)Tch^/*J [*Foxp3^gfp^*; expression of a green fluorescent protein–encoding gene (*gfp*) is controlled by transcriptional control elements of the *Foxp3* (forkhead box protein P3 gene)], C57BL/6-Tg(TcraTcrb)425Cbn/J (OT-II), and B6.SJL-*Ptprc^a^ Pepc^b^*/BoyJ (CD45.1) mice were purchased from Jackson Laboratory (Bar Harbor, ME). The OT-II and CD45.1 strains were crossed to one another. *Tlr4*^–/–^ mice were obtained from S. Akira (Osaka University, Osaka, Japan). Mice were housed at a maximum of three animals per 12.9 in. × 7.5 in. × 5.6 in. polysulfone static Micro-Isolator cage (Lab Products, Seaford, DE) in specific pathogen–free conditions at the National Institute of Environmental Health Sciences (NIEHS). Paper bedding (Diamond Soft; Harlan Teklad, Indianapolis, IN) was used to minimize inhaled dust, and the cages were changed twice weekly. Experiments began when mice were 6–12 weeks of age. All animals were treated humanely and with regard for alleviation of suffering. All animal experiments were conducted in accordance with the NIEHS Institutional Animal Care and Use Committee.

*Adoptive transfer of OT-II T cells and analysis of cytokines in regional LNs.* CD4^+^ T cells were prepared as previously described (Whitehead et al. 2011) from CD45.1 × OT-II mice (carrying the CD45.1 allele), which carry a transgene encoding a T-cell receptor specific for OVA. These T cells were transferred by retroorbital injection into C57BL/6J mice (which carry the CD45.2 allele). After 4 hr, recipient mice were sensitized to OVA by inhaled LPS. LPS doses ranged in 100-fold increments and included the previously reported doses of 10 μg and 10^–1^ μg (Eisenbarth et al. 2002; Wilson et al. 2009), as well as much smaller doses, down to 10^–7^ μg. The 0-μg LPS dose (OVA alone) was used as the control. Four days later, mediastinal LNs were excised, minced, pressed through a 70-μm strainer, and then the cells were cultured at 5 × 10^5^ in 100 μL RPMI media with 10% fetal bovine serum and 10 μg/mL OVA for 2 days. Culture supernatants were analyzed for the cytokines IL-4, IL-5, IL-17A, and interferon-γ (IFN-γ) using a multiplexed fluorescent bead–based immunoassay (Bio-Plex; Bio-Rad laboratories, Hercules, CA) according to the manufacturer’s instructions.

*House dust extracts (HDEs)*. HDEs were prepared as described previously by Sever et al. (2007). Endotoxin levels were assayed by a Limulus amebocyte lysate assay (Lonza, Karlsruhe, Germany), and allergens were measured using a multiplex array for indoor allergens (MARIA; Indoor Biotechnologies, Charlottesville, VA), according to the manufacturer’s instructions.

We evaluated two HDEs, both containing dust mite allergens but with different endotoxin activity. One of these HDEs had endotoxin activity of 2,000 endotoxin units (EU)/mL, approximately equal to 10^–2^ μg LPS/20 μL HDE (Endo_lo_). The other HDE had a higher endotoxin activity, approximately equal to 10^–1^ μg LPS/20 μL HDE (Endo_mod_).

We determined the most effective concentration of HDEs for promoting allergic responses to inhaled OVA by performing a dose–response experiment. We instilled C57BL/6J mice with 100 μg OVA together with 1, 5, or 20 μL HDE. Following OVA challenge (described below), the extent of airway inflammation was determined.

*LPS- and HDE-mediated allergic sensitization and challenges.* In the mornings of days 0 and 7, C57BL/6J and *Tlr^–/–^* mice were anesthetized with inhaled isoflurane and sensitized by oropharyngeal instillation (Wilson et al. 2009) of LPS-depleted OVA (catalog no. 321000; BioVendor Inc., Candler, NC) alone or together with various amounts of *Escherichia coli* LPS (catalog no. L2630; Sigma Chemical Co., St. Louis, MO), HDEs, or LPS-supplemented HDEs. Where indicated, negative control mice received instillations of phosphate-buffered saline (PBS). Asthma-like responses were elicited by exposing sensitized animals to nebulized (Ultra-Neb99; DeVilbiss Healthcare, Somerset, PA) 1% OVA (catalog no. A5503; Sigma) in saline, either for 1 hr on a single occasion (day 14) or for 30 min on 6 consecutive days (days 14–19). Using separate groups of C57BL/6J mice, we measured peak cellular inflammation and AHR 48 hr after a single challenge and 24 hr after multiple challenges.

*AHR analysis.* For AHR evaluation, mice were anesthetized by intraperitoneal (ip) injection with urethane (1.5 g/kg), paralyzed by ip injection with pancuronium bromide (0.8 mg/kg), and intubated. AHR was analyzed using the FlexiVent mechanical ventilator system (Scireq; Montreal, Quebec, Canada) as previously described (Wilson et al. 2009).

We used the single-compartment model of the lung to assess total respiratory system resistance after delivery of aerosolized methacholine (0–50 mg/mL in C57BL/6J mice; 0–25 mg/mL in BALB/cJ mice) using an ultrasonic nebulizer. We report peak resistance values.

*Antibody response.* To assess antibody responses, venous blood was collected, and serum was separated via plasma separator tubes and centrifugation. To quantify IgE in serum samples, samples (diluted 1:20) were analyzed using the murine IgE ELISA kit (BD Biosciences, San Diego, CA).

*Cellular inflammation*. Whole-lung bronchoalveolar lavage (BAL) was performed 4 hr postchallenge. Differential analysis of cells in BAL fluid (BALF) was performed as described previously (Hollingsworth et al. 2004). For cytokine analysis, IL-4, IL-5, IL-17A, and IFN-γ were measured in BALF using a multiplexed fluorescent bead-based immunoassay as described above.

*Flow cytometry*. For analysis of OVA-specific Treg cells in the lung, mice that received adoptive transfers of OT-II cells were sensitized with OVA together with various amounts of LPS and challenged on days 7–12 with 1% OVA aerosol for 30 min. On day 13, lungs were excised, minced, and enzymatically digested as previously described (Nakano et al. 2011). Cells were diluted to 2 × 10^6^/100 μL and incubated with a nonspecific binding blocking reagent cocktail of anti-mouse CD16/CD32 (2.4G2), normal mouse, and normal rat serum (Jackson ImmunoResearch, West Grove, PA) for 5 min, then stained with fluorochrome-labeled antibodies against mouse CD4 (RM4-5), CD45.1 (A20), Foxp3 (FJK-16s), and ICOS (inducible T-cell costimulator; C398.4A) (eBioscience, San Diego, CA). Tregs positive for both CD4 and FOXP3 were then incubated with antibodies against ICOS (Wilson et al. 2009). Cells were evaluated using an LSR II flow cytometer (BD Biosciences, San Jose, CA), and data were analyzed using FlowJo software, version 9.6 (Treestar, Ashland, OR).

*Statistics*. Statistical differences between multiple groups were first calculated using one-way analysis of variance. We used a post hoc Dunnett’s multiple comparison test to compare mice treated with OVA plus different doses of LPS with control mice treated with OVA alone. To compare mice treated with OVA plus 10^–1^ μg LPS with mice treated with lower LPS doses, we used post hoc Student’s *t*-test. We used Student’s *t*-test when comparing only two groups. Analyses were performed using GraphPad Prism, version 5.02 (GraphPad Software Inc., San Diego, CA). *p*-Values < 0.05 were considered statistically significant.

## Results

**Figure 1:**
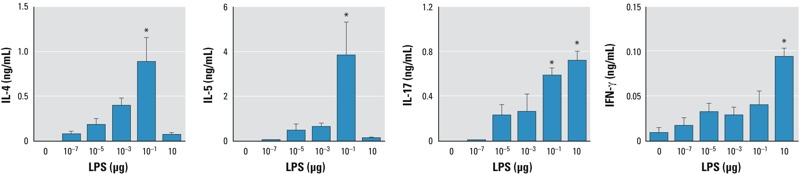
Effect of LPS on cytokine production in cultured LNs from C57BL/6J recipients of OVA-specific T cells. Mice were sensitized to OVA using LPS as adjuvant, and the cytokines IL‑4, IL‑5, IL‑6, and IFN‑γ were measured in cultured mediastinal LNs collected 24 hr postsensitization. Values shown are mean ± SE and represent data from one of two experiments yielding similar results (*n* = 6 mice/group). *p < 0.05 compared with 0 μg LPS (OVA-only controls).

*Effect of LPS dose on T-cell priming in draining LNs*. We first investigated the impact of inhaled LPS dose on the initiation of antigen-specific T-cell priming, which occurs in regional LNs. C57BL/6 mice retroorbitally injected with OVA-specific T cells from OT-II mice (CD45.1) were exposed via inhalation to highly purified OVA together with various amounts of LPS (0–10 μg). After incubating cells from excised regional LNs with OVA, the culture supernatants were analyzed for signature cytokines of different T helper cell lineages. The concentration of the Th2 cytokines IL-4 and IL-5 in these supernatants increased in concert with the dose of LPS used during the sensitization, except for the highest dose of LPS (10 μg); with this dose, almost no IL-4 or IL-5 was detected (Figure 1). IL-17 production in regional LNs also increased with the dose of LPS used, including 10 μg LPS. The concentrations of IFN-γ also increased with the amount of inhaled LPS, including the 10-μg dose, in agreement with previous reports that high doses of LPS prime Th1 responses (Eisenbarth et al. 2002).

**Figure 2:**
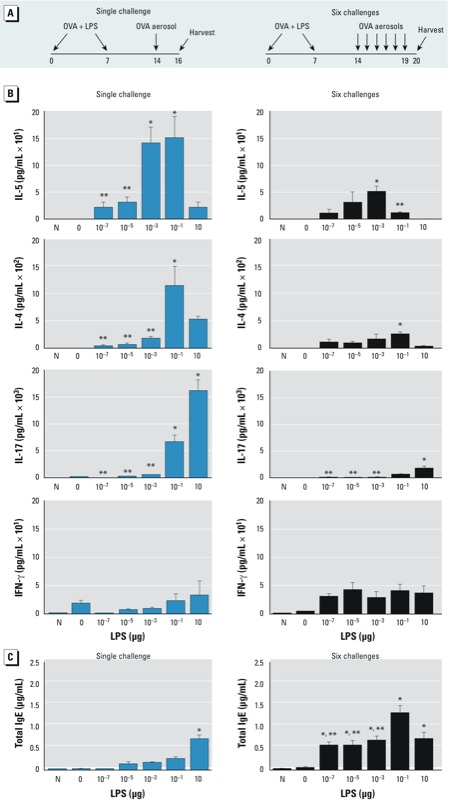
Effect of inhaled LPS dose on cytokine production in the lung following allergen challenge of C57BL/6 mice. (*A*) Time line of sensitization, challenge(s), and harvest. (*B,C*) Naïve (previously untreated) mice (N) and mice sensitized to OVA using LPS were challenged to aerosolized OVA on a single occasion or on 6 consecutive days, and BALF and sera were collected for analysis of cytokines and IgE, respectively. (*B*) Mean cytokine concentrations in BALF (limits of detection: 2.1 pg/mL for IL‑4, 0.3 pg/mL for IL‑5, 0.8 pg/‌mL for IL‑17, and 1.2 pg/mL for IFN-γ). (*C*) Total serum IgE (limit of detection, 0.016 μg/mL). Values shown in (*B*) and (*C*) are mean ± SE and respresent data from one of two independent experiments yielding similar results (*n* = 6 mice/group). *p < 0.05 compared with 0 μg LPS (OVA-only controls). **p < 0.05 for lower LPS dose compared with 10^–1^ μg LPS.

*Impact of LPS dose on cytokines in the lung and on serum IgE*. We next analyzed BALF to determine whether the production of cytokines in the lung after OVA sensitization and challenge would follow the same trend as those produced in regional LNs after sensitization. BALF was collected from mice sensitized to OVA using various doses of LPS and subsequently exposed to aerosolized OVA either on a single occasion to mimic intermittent allergen exposures or on 6 consecutive days to mimic more chronic exposures (Figure 2A). After a single challenge, mice previously administered OVA alone or OVA plus the very high LPS dose (10 μg) had very low concentrations of IL-5 in the airway (Figure 2B), consistent with the low concentrations of this cytokine we observed in regional LNs of similarly sensitized mice (Figure 1). Mice sensitized with either 10^–3^ μg or 10^–1^ μg LPS had much higher concentrations of IL-5 in the airway after OVA challenge, again reflecting the increased amounts of this cytokine seen in regional LNs. After 6 challenges, the concentrations of IL-5 in BALF were lower than after a single challenge, and the trend generally correlated well with the concentrations we had observed in regional LNs. One exception to this trend was mice that had been sensitized to OVA by 10^–1^ μg LPS and challenged on six occasions. Despite having very high levels of IL-5 in regional LNs and in the airway after a single challenge, these animals had lower concentrations of IL-5 in the airway than mice sensitized using lower doses of LPS.

The serum concentration of IL-4, a Th2 cytokine that promotes immunoglobulin class switching to IgE, was lower after multiple challenges than after a single challenge, but it was not lower in mice sensitized with 10^–1^ μg LPS than in those sensitized with 10^–3^ μg LPS, indicating that IL-4 and IL-5 are differentially regulated.

We analyzed production of IL-17 in the airways of challenged mice because it can promote neutrophil recruitment and AHR in this murine model of asthma (Wilson et al. 2009). As we expected, almost no IL-17 was observed in mice that inhaled OVA without LPS. However, the concentrations of this cytokine in BALF increased in proportion to the LPS dose used during sensitization. After six challenges, IL-17 levels were lower than those after a single challenge, but the trend of increased IL-17 with increasing LPS during sensitization was maintained. Thus, unlike IL-5, the concentation of IL-17 in the lung after challenge was closely associated with levels of that cytokine in draining LNs across the entire range of LPS doses tested. IFN-γ, the signature cytokine of Th1 cells, was not significantly different among the various LPS dose groups, suggesting that at these doses, increased Th1 responses do not account for the observed reduction in Th2 responses.

Atopic diseases are associated with elevated levels of IgE antibodies, which bind to Fc receptors on the surface of several cell types, including mast cells and basophils. We therefore studied total serum IgE in mice that had been sensitized to OVA by LPS. We found that titers of this isotype increased in concert with the LPS dose used during sensitization (Figure 2C). The exception to this pattern was mice that had been sensitized using 10 μg LPS and challenged on six occasions. These animals trended toward lower IgE levels than similarly challenged animals that had been sensitized with 10^–1^ μg LPS.

**Figure 3:**
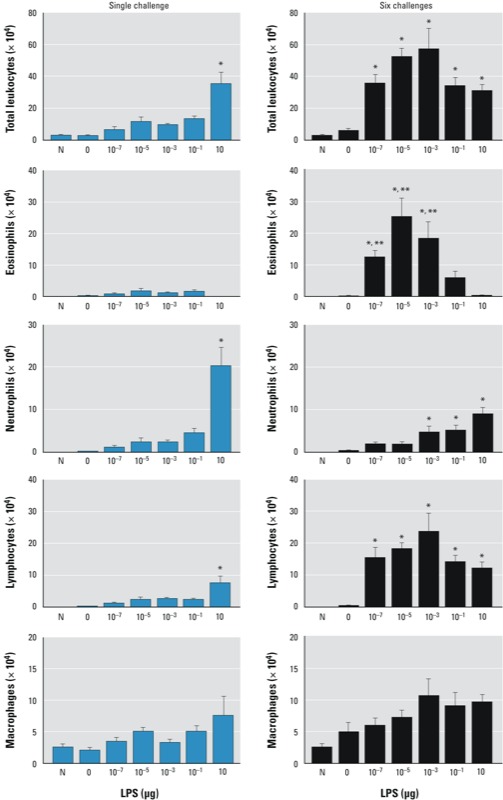
Effect of inhaled LPS dose on leukocyte subset recruitment to the lung following allergen challenge of C57BL/6 mice. Naïve (previously untreated) mice (N) and mice sensitized to OVA using LPS were challenged to aerosolized OVA on a single occasion or on 6 consecutive days; leukocytes were counted in BALF. Values shown are mean ± SE cell number from a single experiment representative of three independent experiments yielding similar results (*n* = 6 mice/group). *p < 0.05 compared with 0 μg LPS (OVA-only controls). **p < 0.05 for lower LPS dose compared with 10^–1^ μg LPS.

*LPS dose during allergic sensitization determines the nature of allergen-induced pulmonary inflammation*. When we examined the impact of LPS dose during sensitization on the types of leukocytes that accumulate in the airway following OVA challenge either on a single occasion or on 6 consecutive days, we observed that C57BL/6J mice that inhaled OVA alone (Figure 3) or LPS alone (data not shown) had very few inflammatory cells in the airway after a single OVA challenge. This suggests that levels of LPS in the home cages were not sufficient to act as a strong adjuvant for OVA sensitization. However, mice receiving OVA together with as little as 10^–7^ μg LPS developed low but measurable airway eosinophilia after a single challenge; this response was strongly increased after six daily challenges (Figure 3). Surprisingly, we observed the strongest eosinophilic response in mice that had been sensitized with 10^–5^ μg LPS. Mice sensitized to OVA with 10^–1^ μg LPS had markedly fewer eosinophils than mice sensitized with lower doses of LPS, despite having the highest amounts of IL-4 and IL-5 in lung-draining LNs (Figure 1). Similar results were obtained for BALB/c mice (see Supplemental Material, Figure S1), ruling out the possibility that our findings were unique to C57BL/6J mice. In animals sensitized to OVA by 10^–1^ μg LPS and challenged by OVA six times, the low number of eosinophils (Figure 3) was consistent with the low concentration of IL-5 in the airways (Figure 2B). Together, these observations suggest that although very low doses of LPS induce relatively weak Th2 responses in the draining LN, they lead to persistent airway eosinophilia, whereas the moderate dose of 10^–1^ μg LPS initially triggers stronger Th2 responses but results in shorter-lived pulmonary eosinophilia.

In contrast to our findings for eosinophil accumulation, the number of neutrophils in the airway following a single OVA challenge was proportional to the amount of LPS administered during OVA sensitization, and were not markedly increased after six OVA challenges. Generally, the number of neutrophils correlated well with airway levels of IL-17. Interestingly, although LPS during sensitization was required for lymphocyte infiltration after OVA challenge, higher LPS doses did not lead to increased lymphocytes. By contrast higher amounts of LPS during sensitization generally led to more macrophages in the airway.

**Figure 4:**
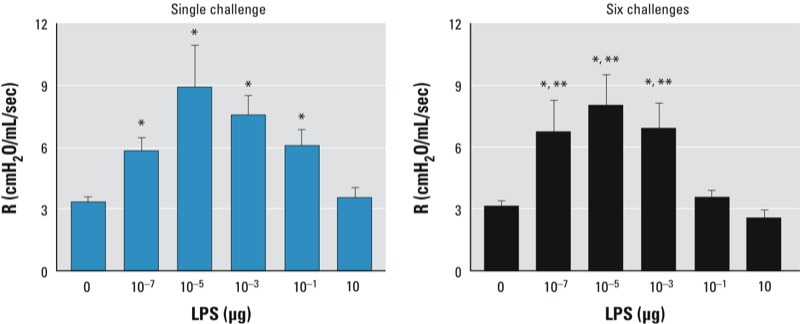
Effect of inhaled LPS dose on airway response to subsequent allergen challenge. Airway resistance (R; cmH_2_0/mL/sec) was measured in anesthetized C57BL/6J mice that were sensitized to OVA using LPS, challenged with aerosolized OVA on a single occasion or on 6 consecutive days, and administered aerosolized methacholine (0–50 mg/mL). Values shown are peak R values (mean ± SE) and represent one of two experiments yielding similar results (*n* = 6 mice/group). *p < 0.05 compared with 0 μg LPS (OVA-only controls). **p < 0.05 for lower LPS dose compared with 10^–1^ μg LPS.

*Effect of LPS dose on subsequent OVA challenge–induced AHR*. AHR is a cardinal feature of asthma. Accordingly, we used invasive measurements of airway resistance to study the effect of LPS dose during allergic sensitization on the development and progression of AHR. As expected, mice that received OVA alone during the sensitization phase did not develop AHR after a single OVA challenge or after six challenges. By contrast, mice sensitized using doses of LPS ranging from 10^–7^ to 10^–1^ μg displayed increased airway resistance in response to methacholine after a single OVA challenge (Figure 4; see also Supplemental Material, Figure S2A). This AHR was sustained in mice that had been sensitized by very low doses of LPS but not in mice sensitized by 10^–1^ μg LPS. Similar results were obtained when BALB/c mice were examined (see Supplemental Material, Figure S2B). Thus, like eosinophilic inflammation, AHR was also sustained after multiple OVA challenges in mice sensitized with very low doses of LPS but not in mice sensitized by the higher, but still moderate, dose of 10^–1^ μg LPS.

**Figure 5:**
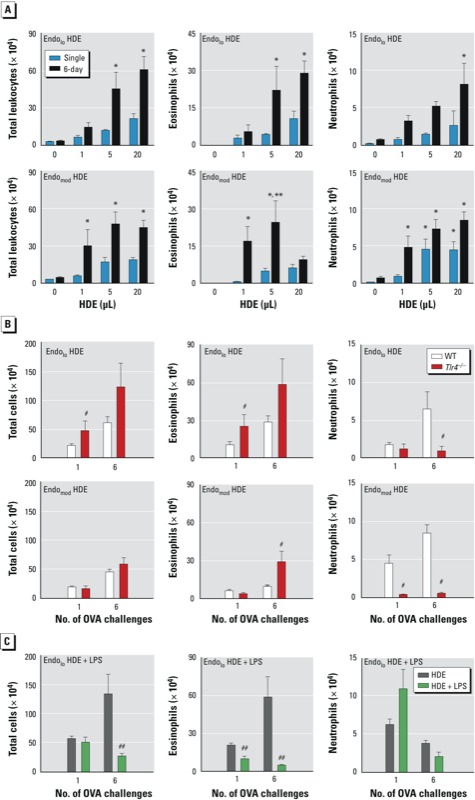
Effect of LPS on induction of effector and regulatory responses by HDEs. Values are shown as the mean ± SE cell number of total leukocytes, eosinophils, and neutrophils in BALF. (*A,B*) Mice were sensitized with OVA together with Endo_lo_ or Endo_mod_ HDEs and challenged with OVA on a single occasion or on six occasions. (*A*) Dose response for Endo_lo_ or Endo_mod_ HDEs in C57BL/6J mice. (*B*) Contribution of LPS component of HDEs to airway leukocytes in *Tlr4*-deficient (*Tlr4*^–/–^) and wild-type (WT C57BL/6J) mice sensitized to OVA using 20 μL of HDE and then challenged as described above. Data in (*A*) and (*B*) are from one of two experiments yielding similar results (*n* = 6 mice/group). (*C*) C57BL/6J mice were sensitized to OVA using Endo_lo_ HDE alone or supplemented with LPS and challenged as described above. Eosinophilic inflammation was inhibited in HDE plus LPS mice. Data are from a single experiment (*n* = 6 mice/group). *p < 0.05 compared with 0 μL HDE (OVA-only controls). **p < 0.05 for 20 μL compared with the lower HDE dose. ^#^*p* < 0.05 compared with WT. ^##^*p* < 0.05 compared with HDE alone.

*Environmental amounts of LPS are sufficient to prime both Th17 and regulatory responses to inhaled allergens.* We examined whether the amounts of LPS found in natural environments are capable of priming Th2 and Th17 responses that lead to airway eosinophilia, neutrophilia, and AHR, reasoning that common house dust would provide a good representation of indoor environments. We therefore prepared extracts from dust samples and tested their abilities to promote allergic responses to co-instilled OVA. We evaluated two HDEs that contained dust mite allergens but had different endotoxin activity (see Supplemental Material, Figure S3A). Endo_lo_ had a relatively low endotoxin activity (approximately equal to 10^–2^ μg LPS/20 μL HDE), and Endo_mod_ had a higher endotoxin activity (approximately equal to 10^–1^ μg LPS/20 μL HDE). In a dose–response experiment in C57BL/6J mice, we found that after OVA challenge, neutrophilic and eosinophilic responses generally increased with increasing doses of HDE, and that at low doses, the Endo_mod_ HDE was more effective than Endo_lo_ (Figure 5A). However, mice that were sensitized with 20 μL of Endo_mod_ HDE had fewer airway eosinophils after the 6-day challenge than did mice sensitized using lower doses of this HDE. This observation was reminiscent of the reduced airway eosinophilia observed in mice exposed to 10^–1^ μg LPS and challenged six times compared with mice exposed to lower doses of LPS (Figure 3).

HDEs are complex mixtures and typically contain multiple adjuvants and allergens (see Supplemental Material, Figure S3B). To confirm that moderate amounts of LPS in house dust can reduce Th2 responses and eosinophilic inflammation, we carried out two additional experiments. First, we studied the Endo_mod_ HDE in *Tlr4*-deficient mice, which are unable to respond to LPS. These *Tlr4*-deficient mice displayed very little neutrophilic inflammation after OVA challenge, but they had increased numbers of eosinophils (Figure 5B). This result suggests that LPS residing in HDE promotes adaptive immune responses that drive neutrophil accumulation in the lung and suppress eosinophil accumulation. To confirm this, we added 10^–1^ μg of exogenous LPS to the Endo_lo_ HDE and tested its adjuvant activity. This addition of LPS led to significantly reduced eosinophils in the airway, particularly in mice that had been challenged on six occasions (Figure 5C).

**Figure 6:**
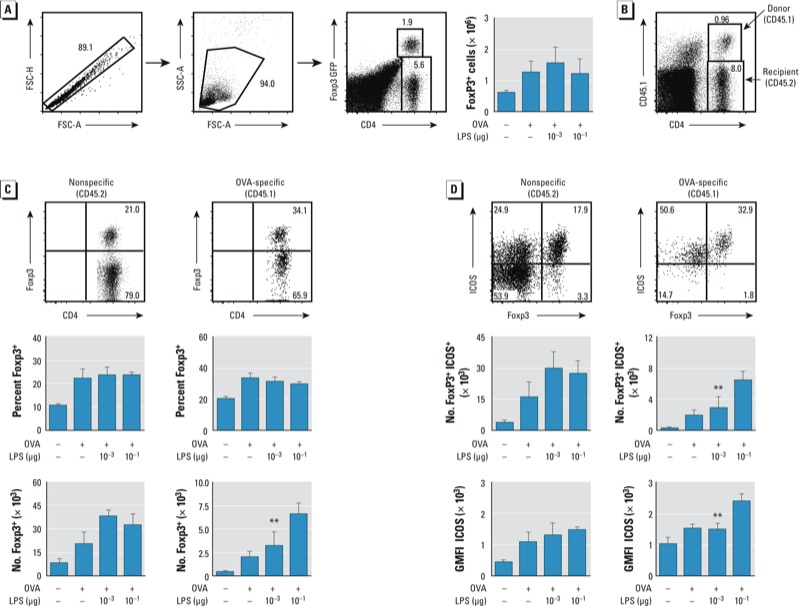
Analysis of Tregs in *Foxp3^gfp^* mice. Abbreviations: FSC, forward scatter; GMFI, geometric mean of fluorescent intensity; SSC, side scatter. (*A*) Gating strategy used to identify CD4^+^ Foxp3^+^ Tregs, as well as total numbers of Tregs in mice sensitized to OVA using the LPS and subsequently challenged with OVA on 6 consecutive days (right). (*B*) Gating strategy for OVA-specific OT‑II (CD45.1) and nonspecific (CD45.2) Foxp3^+^ Tregs. (*C*) Gating for nonspecific (left) and OVA-specific (right) Foxp3+ cells (top) and the percent (middle) and number (bottom) of CD4^+^ T cells that express Foxp3. (*D*) ICOS display on nonspecific (left) and OVA-specific (right) Foxp3^+^ Tregs. Gating for ICOS^+^Foxp3^+^ cells (top), numbers of CD4^+^ Foxp3^+^ cells displaying ICOS (middle), and GMFI (and SE) for ICOS staining (GMFI ICOS; bottom). Data are from one of two experiments yielding similar results (*n* = 4–5 mice/group for all experiments). **p < 0.05 for 10^–3^ μg LPS compared with 10^–1^ μg LPS.

*Effect of LPS dose during sensitization on OVA-specific and nonspecific Tregs in the chronic asthma model*. We next investigated mechanisms that might be responsible for the reduction in eosinophils observed after multiple exposures to OVA in mice sensitized using moderate amounts of LPS. Because pulmonary levels of IFN-γ were not significantly increased in mice sensitized with 10^–1^ μg LPS compared with those sensitized using lower LPS doses, it seemed unlikely that Th1 responses were entirely responsible for suppressing eosinophilic inflammation. Moreover, analysis of draining LNs revealed that Th2 cytokines were highest when 10^–1^ μg LPS was used during sensitization. We considered the possibility that inhaled LPS induces regulatory responses that gain in strength after multiple challenges and suppress eosinophilic inflammation. In some murine models of asthma, IL-10 can suppress allergic inflammation in the lung (Joetham et al. 2007; Kearley et al. 2005; Oh et al. 2002), but amounts of this cytokine were similar in lungs of mice sensitized using high or low doses of LPS (data not shown). We therefore studied Foxp3^+^ CD4^+^ Tregs, which can effectively suppress established allergic responses (Lloyd and Hawrylowicz 2009), including those induced by OVA/LPS (Whitehead et al. 2011). Two main types of Tregs have been identified: natural (n) Tregs that develop in the thymus and recognize self-antigens, and induced (i) Tregs that develop in the periphery and recognize exogenous antigens. These two cell types have overlapping but distinct activities and can be distinguished by their transcriptional profile (Bilate and Lafaille 2012). When we evaluated total Tregs in the lung using *Foxp3^gfp^* mice, we found that total GFP^+^ CD4^+^ Tregs were present at similar levels in the lung whether 10^–3^ μg LPS or 10^–1^ μg LPS was used in the sensitization phase (Figure 6A). These experiments ruled out total Treg cell number as being the sole factor responsible for suppression, but left open the possibility that allergen-specific iTregs might be particularly important in this regard. To investigate this possibility, we adoptively transferred OVA-specific OT-II (CD45.1) cells into recipient mice (C57BL/6J; CD45.2) prior to LPS sensitization and OVA challenge on 6 consecutive days. Recipient CD4^+^ T cells comprised the vast majority of total CD4^+^ T cells and their numbers were increased in OVA/LPS-sensitized mice (Figure 6B) compared with mice treated with PBS or OVA alone (data not shown). The number and percentage of recipient Foxp3^+^ Tregs were similar in mice sensitized using 10^–3^ μg LPS or 10^–1^ μg LPS (Figure 6C), but OVA-specific CD45.1 CD4^+^ donor T cells, including Foxp3^+^ CD4^+^ Tregs, were more abundant in mice sensitized using 10^–1^ μg LPS than in those sensitized using 10^–3^ μg LPS (Figure 6C). This suggested that inhalation of 10^–1^ μg LPS might, after multiple OVA challenges, lead to suppression of allergic responses by increasing the number of OVA-specific Foxp3^+^ CD4^+^ iTregs in the lung.

Tregs are heterogeneous cells that might have functional differences. For example, Foxp3^+^ CD4^+^ nTregs in humans are composed of populations with distinct cell surface display levels of ICOS (Ito et al. 2008). Previous studies have shown that suppression of airway inflammation and AHR is associated with high levels of ICOS expression on CD4^+^ Tregs (Whitehead et al. 2011) and that adoptive transfer of ICOS^+^ CD4^+^ T cells, but not ICOS^–^ cells, suppresses established AHR in mice (Shalaby et al. 2012). We therefore compared numbers of ICOS^+^ Foxp3^+^ Tregs, as well as the amount of ICOS on the cell surface of Tregs, in mice sensitized using 10^–3^ or 10^–1^ μg LPS. The total numbers of recipient ICOS^+^ Foxp3^+^ Tregs were similar in mice from these two groups (Figure 6D). However, the number of OVA-specific donor ICOS^+^ Foxp3^+^ Tregs, as well as the intensity of ICOS staining on these cells, was higher in mice sensitized using the suppressive dose of LPS (10^–1^ μg) than in those sensitized with the nonsuppressive dose (10^–3^ μg). This suggests that moderate doses of inhaled LPS might lead to suppression of established allergic responses in the chronic challenge model by increasing the number of ICOS^+^ Foxp3^+^ Tregs and the levels of ICOS on these cells.

## Discussion

It is widely accepted that environmental endotoxins can impact the prevalence and severity of allergic asthma. However, whereas some studies have shown a positive association between endotoxin and asthma (Ross et al. 2000; Thorne et al. 2005), others have suggested that LPS protects against development of this disease (Braun-Fahrländer et al. 2002; El-Sharif et al. 2006; Gehring et al. 2004). In mice, very high doses of inhaled LPS have been previously reported to promote Th1 responses, with lower doses promoting Th2 responses (Eisenbarth et al 2002). However, the impact of LPS levels on Th17 and Tregs has not been extensively studied. This issue has become increasingly important because these two cell types are now thought to have critical roles in some forms of asthma, including noneosinophilic asthma. Findings of the present study shed light on this issue by showing that the nature and longevity of immune responses to inhaled OVA are remarkably sensitive to the amount of LPS inhaled at the time of sensitization. The lowest dose of LPS that we tested (10^–7^ μg) is 1 million times lower than the 10^–1^ μg used previously in this model to elicit allergic sensitization to inhaled OVA (Eisenbarth et al. 2002; Wilson et al. 2009). Although effective even at extremely low concentrations, some LPS was necessary for sensitization because mice receiving OVA with no LPS failed to become sensitized, as judged by their lack of allergic responses to subsequent OVA challenge. It should be noted that our mice are housed in conditions designed to minimize pulmonary inflammation from dust or endotoxin, and it is possible that mice housed in dirtier or dustier conditions might have responded differently.

Reported amounts of endotoxin in the environment vary according to the location, season, and methods used for endotoxin detection, and they depend on whether dust, defined particulate matter, or ambient air is measured. Ambient air contains endotoxin that typically ranges from 0.1 to 4 EU/m^3^ (Tager et al. 2010), although endotoxin levels can reach up to 50,000 EU/m^3^ in some industrial settings, such as swine containment facilities (Dungan 2011). In the experiments described here, experimental mice were instilled with a liquid containing 0.001 EU (10^–7^ μg LPS) to 100,000 EU (10 μg LPS), spanning the range of doses encountered in the environment. The ability of extremely low doses of LPS to prime allergic responses suggests that the amount of this bacterial product found in the environment are sufficient to promote allergic responses to environmental allergens. However, continuous exposure to low amounts of LPS in ambient air is likely quite different than intermittent exposures to higher doses, even if the cumulative doses are similar.

Eisenbarth et al. (2002) reported that 10^–1^ μg of inhaled LPS promotes Th2 responses, whereas the much higher dose of 100 μg LPS promotes Th1 responses. These observations led to the hypothesis that a threshold level of LPS switches Th2 responses to Th1 responses (Vercelli 2003). Results from the present study are in partial agreement with this hypothesis because we found that in draining LNs, the highest level of LPS we used (10 μg) promoted Th1 responses—and Th17 responses—at the expense of Th2 responses. However, our findings also suggest that the relationship between environmentally relevant amounts of LPS and immune responses is much more complex than this. At low LPS doses (≤ 10^–1^ μg), increasing amounts of LPS during sensitization led to increases in Th2 cytokines in draining LNs, as well as increases in Th1 and Th17 cytokines. Thus, in contrast to a widely held belief, environmentally relevant concentrations of LPS do not inhibit the initiation of Th2 responses in draining LNs. However, mice sensitized using the moderate dose of 10^–1^ μg LPS and challenged with OVA six times had markedly less IL-5 and eosinophilia in BALF and lower AHR after six challenges than mice sensitized using lower amounts of LPS. It seems unlikely that this reduction in asthma-like features is due entirely to a switch from Th2 to Th1 responses because we did not observe increased airway IFN-γ in mice sensitized using 10^–1^ μg LPS and because, for doses up to 10^–1^ μg, increasing concentrations of LPS led to stronger Th2 responses in draining LNs. We therefore propose that 10^–1^ μg LPS induces regulatory responses that suppress Th2 and Th17 cell action, particularly after multiple allergen challenges. We have previously shown that the number of Tregs in the lung increases with the number of OVA challenges and that depletion of Foxp3^+^ Tregs increases allergic inflammation and AHR (Whitehead et al. 2011). In the present study, we found that OVA-specific iTregs were more abundant in mice sensitized using the suppressive dose of 10^–1^ μg LPS than in mice receiving the nonsuppressive dose of 10^–3^ μg LPS. This increase in Treg number was partly due to a corresponding increase in total OVA-specific T cells, but a threshold number of Tregs in the lung might be required for suppression after multiple challenges. It is also possible that OVA-specific iTregs, although fewer in number, have a greater impact on pulmonary inflammation than nTregs.

TLR4 is present on the surface of at least some Tregs, and LPS can promote expansion of these cells (Caramalho et al. 2003). However, in the experiments described here, preexisting OVA-specific iTregs would be unlikely because the animals had not previously been exposed to OVA. Thus, the LPS instilled together with OVA during the sensitization phase likely functioned to promote the development of these cells, which probably proliferated during the challenge phase. Cell surface display levels of ICOS on Foxp3^+^ Tregs were also highest in mice sensitized using the suppressive dose of 10^–1^ μg LPS. We did not directly compare the function of Tregs containing high and low levels of ICOS in the present study, but it has been previously observed that ICOS is required for suppression of allergic responses (Whitehead et al. 2011) and that adoptive transfer of ICOS^+^ CD4^+^ T cells suppresses airway eosinophilia and AHR, whereas transfer of ICOS^–^ CD4^+^ T cells does not (Shalaby et al. 2012).

The LPS used in the present study likely contains other microbial products that might have contributed to the observed immune and inflammatory responses. Similarly, the composition of HDEs is also highly complex and undoubtedly includes microbial products and allergens in addition to LPS. However, an important advantage of using HDEs is that they are derived from typical indoor environments and therefore more accurately reflect natural exposures than does instillation of arbitrary amounts of any single microbial product. It is therefore important that addition of LPS to the Endo_lo_ HDE was sufficient to dramatically reduce the ability of that extract to promote sustained airway eosinophilia after six OVA challenges.

## Conclusions

Our findings show that immune responses to inhaled allergens are highly dependent on the doses of inhaled LPS, including the amounts found naturally in the environment. We propose that exposure to relatively low doses of LPS promotes classical Th2-driven allergic responses to inhaled allergens, whereas moderate doses of this microbial product induce stronger Th17 responses and associated neutrophilia. In addition, inhalation of moderate doses of LPS during sensitization induces regulatory responses that, after multiple allergen exposures, limit the severity and longevity of asthma-like features.

## Supplemental Material

(418 KB) PDF
