# Supplementary material for: Modulation of Distinct Asthmatic Phenotypes in Mice by Dose-Dependent Inhalation of Microbial Products
Source: Environ Health Perspect. 2013 Oct 29;122(1):34–42. doi: 10.1289/ehp.1307280 (PMC3888577; doi:10.1289/ehp.1307280)
Supplement: (418 KB) PDF [file ehp.1307280.s001.508.pdf]

Environmental Health Perspectives doi: 10.1289/ehp.1307280

Supplemental Material

Modulation of Distinct Asthmatic Phenotypes in Mice by Dose-Dependent Inhalation of Microbial Products

Gregory S. Whitehead, Seddon Y. Thomas, and Donald N. Cook

Table of Contents:

Figure S1. Effect of inhaled LPS dose during allergen sensitization on airway inflammation in BALB/c mice.

Figure S2. Effect of inhaled LPS dose during allergen sensitization on airway reactivity to methacholine (MCH) in C57BL/6 (A) and BALB/c (B) mice.

Figure S3. Endotoxin and allergens in HDEs.

Figure S1

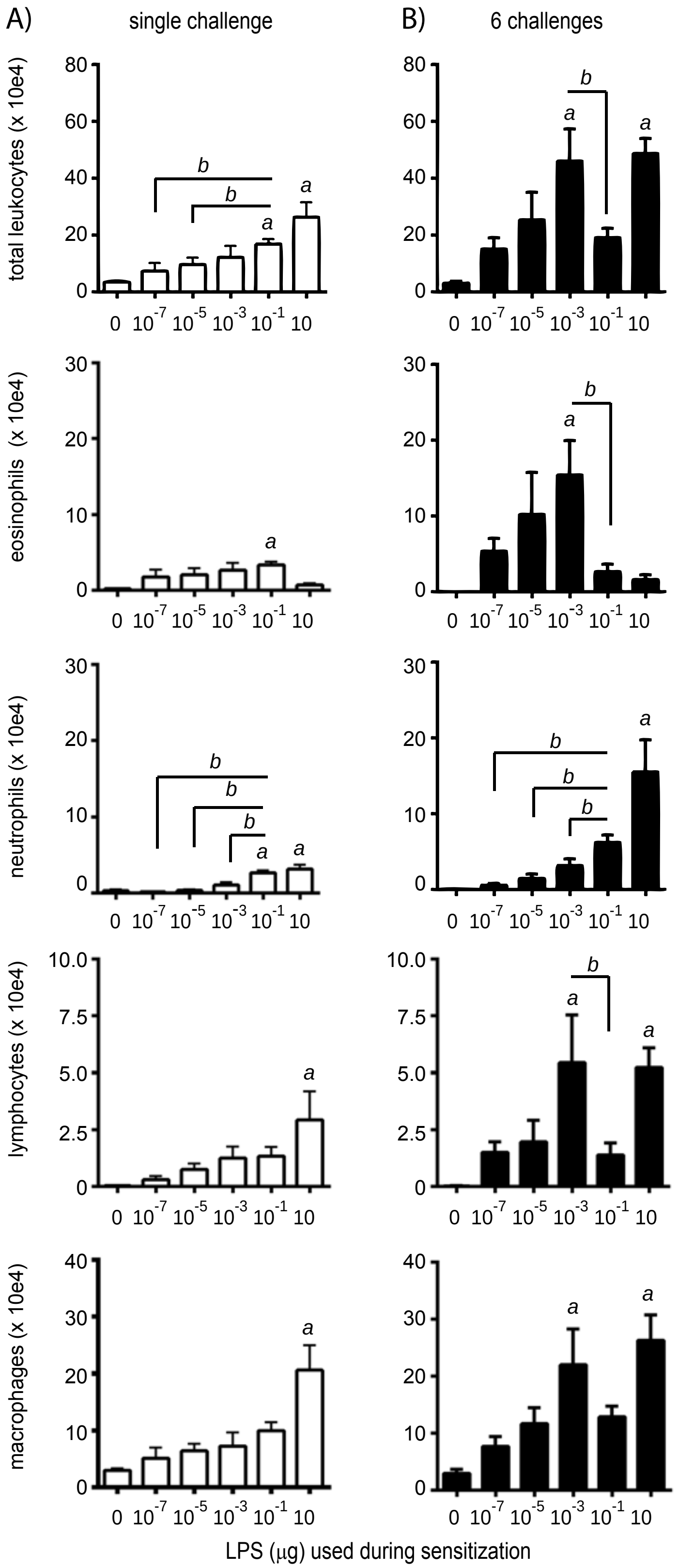

Figure S1. Effect of inhaled LPS dose during allergen sensitization on airway inflammation in BALB/c mice. Mice sensitized to OVA using the indicated amounts of LPS were challenged to aerosolized OVA on a single occasion (A), or on six consecutive days (B). Shown are mean cell numbers ± SEM of the indicated leukocyte subsets in BAL 48 h after the last challenge. Data are from one of 2 independent experiments giving similar results. n = 6 mice/group. *a*, $P < 0.05$, OVA-LPS sensitized mice vs. 'OVA only' controls; *b*, $P < 0.05$, 10^{-1} μg LPS vs. lower dose LPS groups.

Figure S2

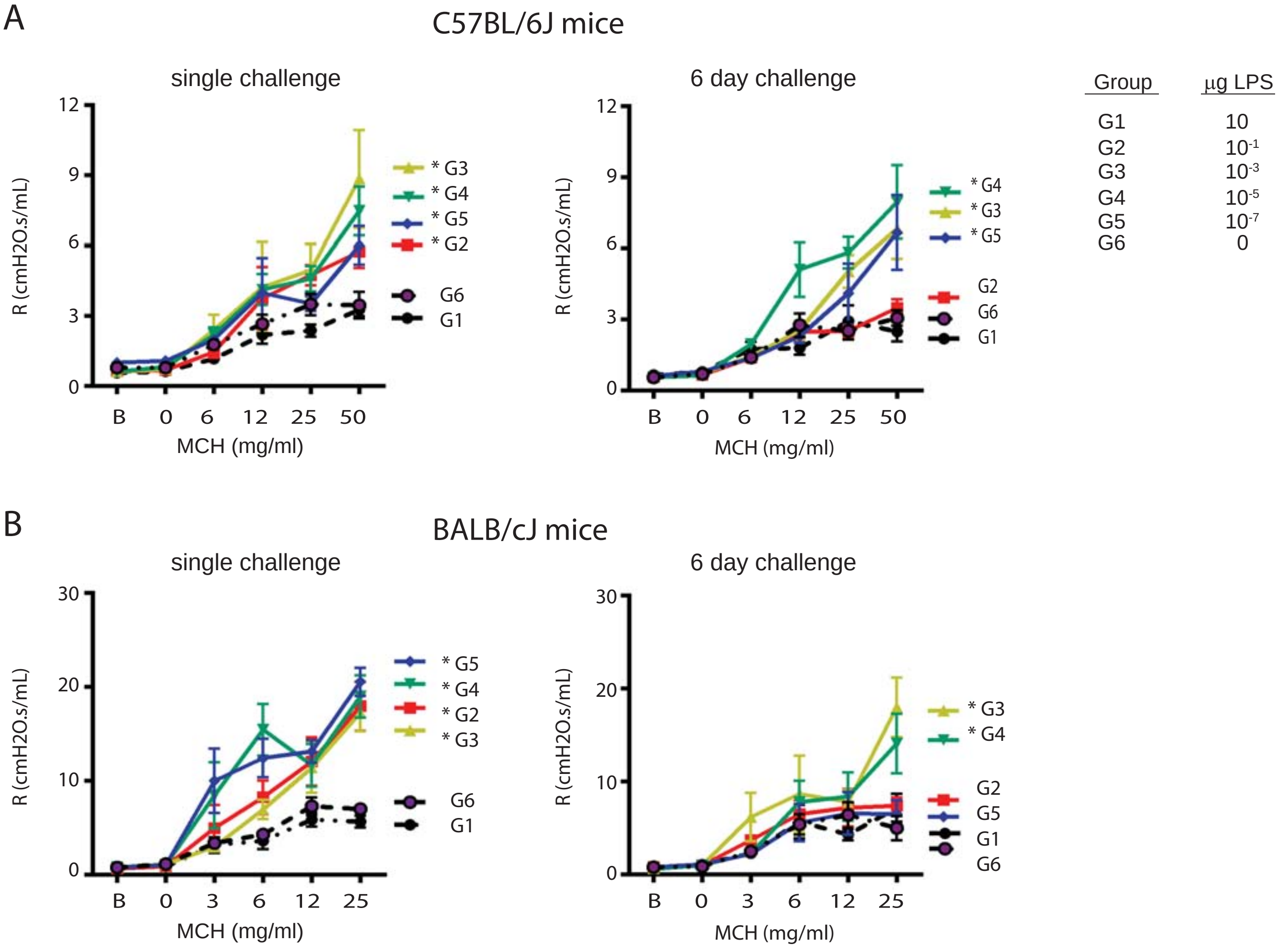

Figure S2. Effect of inhaled LPS dose during allergen sensitization on airway reactivity to methacholine (MCH) in C57BL/6 (A) and BALB/c (B) mice. Airway resistance (R) was measured in mice sensitized to OVA together with the amount of LPS indicated for each group, and then subsequently challenged with aerosolized OVA on a single occasion or on 6 consecutive days. Shown are the mean R values ± SEM from peak responses at each dose of MCH. The data from C57BL/6 mice are from one of 2 independent experiments giving similar results; the data from the BALB/c mice are from a single experiment. n = 6 mice/group. * $P < 0.05$; OVA-LPS sensitized mice vs. ‘OVA only’ controls.

Figure S3

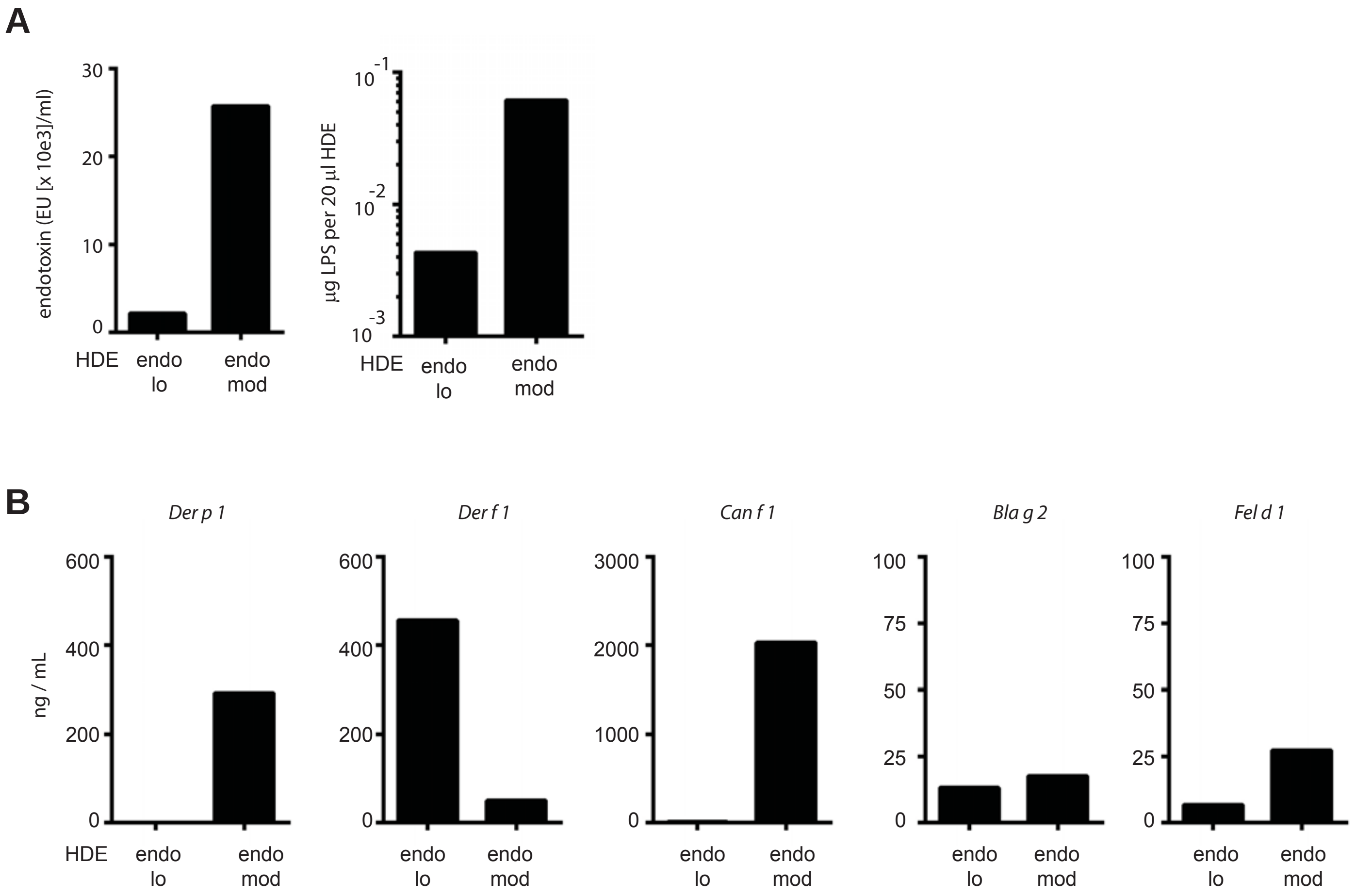

Figure S3. Endotoxin and allergens in HDEs. (A) The number of endotoxin units (EU) per ml of each HDE is shown, as well as a LPS mass equivalent (µg) for a 20 µl sample of each HDE. (B) Allergen content of HDEs. Allergens assayed include those derived from the house dust mites *Dermataphogoides pteronyssinus* (*Der* p 1) and *Dermataphogoides farina* (*Der* f 1), dog *Canus familiarus* (*Can* f 1), German cockroach *Blatella Germanica* (*Bla* g 2), and cat *Felis catus* (*Fel* d 1).
